# Do French Consumers Have the Same Social Representations of Pulses as Food Industry Professionals?

**DOI:** 10.3390/foods9020147

**Published:** 2020-02-01

**Authors:** Juliana Melendrez-Ruiz, Gaëlle Arvisenet, Vincent Laugel, Stéphanie Chambaron, Sandrine Monnery-Patris

**Affiliations:** Centre des Sciences du Goût et de l’Alimentation, AgroSup Dijon, CNRS, INRAE, Université Bourgogne Franche-Comté, F-21000 Dijon, France; gaelle.arvisenet@agrosupdijon.fr (G.A.); laugelvin@gmail.com (V.L.); stephanie.chambaron-ginhac@inra.fr (S.C.); sandrine.monnery-patris@inra.fr (S.M.-P.)

**Keywords:** pulses, social representations, structural approach, pulse food industry, supply chain, professionals, word association

## Abstract

Pulses present many advantages for human health, nutrition, sustainability, and the environment. Despite efforts in recent years by the pulse industry and national authorities to favor pulses, consumption in France remains relatively low, at 1.7 kg/per person in 2016, compared to 1920 when it was around 7.2 kg/per person. To understand social representations of pulses in France, 80 French nonvegetarian consumers and 35 professionals from the pulse industry were asked to say five words spontaneously evoked by the inductor “pulses”. They then had to rank these five words in order of importance and rate their valence. The structural approach was used to analyze social representations for each group independently. Our results highlight differences in the structure and content of social representations for pulses. Consumer responses suggested only vague impressions of pulses, but taste evocations were nevertheless rated positively. By contrast, professionals tended to focus specifically on protein content and culinary preparation. These differences could explain some barriers to pulse consumption, and improved communication should be a key target. Efficient communication must consider the concepts most frequently used by consumers when referring to pulses, and those ranked as most important.

## 1. Introduction

There is growing interest worldwide in sustainable diets, and pulses present many advantages: They have a positive impact on environmental sustainability, on human nutrition and health, and on food security [1]. Despite their numerous interesting properties, however, pulses have become less popular in France over the last century [2,3], with annual consumption falling to 1.7 kg per person in 2016 [4]. The lack of technology to create new, attractive, pulse-based food products has been put forward as one of the reasons for the low consumption of pulses in France [5]. As a result, innovative technologies have been developed to propose new products, such as processed lentils (e.g., ready-to-eat dishes in sachets, or boxes) [6], and precooked products combining cereals and pulses (e.g., pasta combining wheat and pulse flour) [7]. In addition, the most recent French public nutritional campaign now provides information about pulses [8], with the aim of increasing pulse consumption in France. Nevertheless, it seems that these efforts have not yet been successful. Are they perhaps not adequately adapted to consumer expectations? 

Two recent studies suggested that the main barriers to pulse consumption in France are linked to consumers’ mental representations of pulses [8,9]. A mental representation is a cognitive product, based on individual mental processes that come from interaction with a person’s environment, allowing the meaning of stimuli to be stored in the memory [10]. It was observed that French consumers considered lentils, beans, and chickpeas as foods for vegetarians, requiring lengthy preparation, that were not much liked, and therefore consumed infrequently [9]. Consumers knew the positive properties of lentils, chickpeas, and beans as products with a high protein content, good for the health, and for the environment. Nonetheless, they tended to use these foods only as a side product in their dishes, with an animal protein product as the central component [11]. 

Another type of barrier may be related to inefficient recommendations regarding pulse consumption, based on terms poorly understood by consumers. Communication campaigns aimed at guiding consumers toward healthier and more sustainable diets often use food group names, such as “pulses” or “legumes”, with which French consumers are not necessarily familiar [12]. One way to explore this assumption is through the theory of social representations. A social representation is constructed from a set of beliefs, opinions, attitudes, and information about a certain object [13]. The meaning of this object is created by a system of social negotiations, within a social group [14]. One of the functions of a social representation is to allow the members of a social group to acquire and integrate knowledge. Social representations also facilitate social communication and the transmission and diffusion of knowledge, while prescribing behavior and practices [13]. 

Social representations have been used to investigate whether there is shared understanding among consumers about concepts such as naturalness [15], hedonic food intake in restaurants [16], wine minerality [14], etc. The study of social representations is also particularly interesting for the study of people’s reactions to new foods, such as edible flowers [17]. Social representations play a role in the transition from “what is disturbing and unknown into something familiar and known” [18] (p. 300). The opposite transition has taken place among consumers in France, in the case of pulses, which have been steadily more neglected, particularly during the twenty-first century. Consumer knowledge of pulses is therefore likely to have declined. The study of social representations may allow us to discover whether consumers share common beliefs, practices, and attitudes in relation to pulses. Comparing the social representations of pulses elicited from consumers with those from professionals in the pulse industry and consumers may provide keys to identify and resolve communication problems, which could in turn stimulate consumption. Interactions between consumers and professionals from the food sector are of vital importance, yet so far, they are not very well documented. One Australian study did focus on the relationship between farmers, food processing businesses, and consumers in relation to plant food beliefs and attitudes [19]. This study shows that industry awareness of consumers’ plant food beliefs is generally misguided. In particular, barriers to plant food consumption expressed by consumers were not identified as such by food industry professionals. The authors underlined the need to increase knowledge sharing between consumers and the plant food industry, by creating stronger links between consumers, farmers, and food processing businesses [19]. 

One way to study social representations is the structural approach, which has been used for decades in social psychology research and applied more recently to the consumer science field to understand consumer behavior. This type of analysis provides access to the meaning that a social group attributes to the object of a social representation [20], organized and structured around a central core, composed of one or more elements [13]. The central core represents the system of values corresponding to the cultural and social norms of the group. It remains stable, thus ensuring continuity in contexts that may move and evolve [13]. In addition to the structural approach, it can also be calculate the polarity index, which is one way of accessing implicit attitudes toward the object of a social representation [21]. Attitudes are among the many variables used to investigate human behavior. They result from previous experience with the product (or a similar product from the same category) and from information obtained via word-of-mouth, advertising, product packaging, and advertising in the media [22]. Attitudes can be formed even in the absence of actual experience with an object [23], based on what consumers have seen or heard about the object. Attitudes lead to favorable or unfavorable evaluations of a specific object. If consumers evaluate a product favorably, the use or consumption of this product may increase, whereas a negative evaluation may act as a barrier to consumption [24]. Attitudes remain dynamic and may therefore change over time.

The aim of the present study is to understand the social representations of pulses among two social groups in France: nonvegetarian consumers and professionals from the pulse industry. The hypothesis was that the social representations of professionals and consumers would have very different structures. These differences could aggravate the mismatch between the pulse products on offer and the needs of the consumer, resulting in ever lower levels of consumption. Understanding the discrepancies between the social representations of these two populations, taken together with any common elements, could provide keys for the development of strategies optimized to increase pulse consumption. 

## 2. Materials and Methods 

### 2.1. Participants 

A total of 80 consumers (27 men and 53 women) and 35 professionals from the pulse industry in France (17 men and 18 women) participated in our study. For consumers, the two inclusion criteria were age, between 25 and 65, and no specific diet (e.g., vegetarian or vegan). Consumers were recruited on a voluntary basis, over a three-week period, from a Cultural and Social Center in Dijon, France. The pulse industry professionals were recruited from among the participants at a congress held in 2018, “2èmes Rencontres Francophones des Légumineuses (RFL2)”. The study was carried out several weeks after the congress. Additional pulse industry professionals were recruited through contacts provided by participants and from web-based sources such as “LinkedIn”. For professionals, the only inclusion criterion was to be working in a field related to the pulse supply chain. The age range for professionals was between 30 and 65 years old. The professionals who participated in our study were either experts in pulse seed selection or employed in R&D for industries that transform pulses or farmers growing at least one type of pulses (belonging either to farming cooperatives or interprofessional associations), or store managers. In addition to the three main sectors thus represented in this study (agriculture, processing, and distribution), the supply chain also includes suppliers, transporters, and warehouse managers [25,26]. 

### 2.2. Procedure 

The study was conducted in accordance with the Declaration of Helsinki and was approved by the ethical committee of INSERM N°18-506. Institutional Review Board INSERM (CEEI/IRB) (IRB00003888, IORG0003254, FWA00005831).

Consumers and professionals who agreed to take part in the study were asked to read and fill out a consent form. Consumers received this form from the experimenter just before a face-to-face individual interview. Professionals from the pulse industry were first contacted by email and then received the consent form by email, with an appointment for a phone interview. For both consumers and professionals, the interview lasted from 10 to 15 min.

The experiment started with an evocation task, followed by a ranking and polarity test. A pretest was carried out with the inductor word “car”, in order to ensure that participants understood the task. They were then given the inductor “pulses” (“*légumes secs*” in French). Participants had to say out loud five words or expressions that came spontaneously to mind when they heard the inductor. Once participants had given five words, they were asked to rank each of their words according to relative importance, from 1 to 5 (1 for the word that participants considered the most important and 5 for the least important). Finally, participants had to rate the valence of each of their words, by giving a score from −2 to +2: very negative (−2), negative (−1), neutral (0), positive (+1) or very positive (+2). 

### 2.3. Analysis 

All the words evoked by consumers and by professionals were analyzed qualitatively. A thematic analysis followed the phases proposed by Braun and Clarke [27]: familiarization with the data, generation of initial codes, searching for themes, reviewing themes, and defining/naming the themes. For this last step, a triangulation process was carried out [28]. Three researchers independently sorted all the words by meaning into groups based on meaning, then these three lists of word groups were compared, and the final list of word groups was validated consensually for each set of participants. The resulting word groups were translated into English using a two-way translation process.

#### 2.3.1. Structural Approach

Each word group was then analyzed using the method proposed by Abric [29], also known as the structural approach, which is divided into two main phases. 

For each set of participants, we calculated the total frequency of occurrence and the mean importance score for all the words in a given word group. Cutoff points were then determined for frequency and for importance. The frequency cutoff point was obtained by halving the total frequency of occurrence for the word group with the highest frequency score, while the importance cutoff point was calculated by averaging mean importance scores over all word groups [29]. 

A social representation map was then built for each set of participants, with four zones (2 × 2) delimited by the cutoff points:

- The first zone is the *central core*, with the most frequently cited and most important word groups.
- The second zone is the *first periphery*, where the most frequently cited but less important word groups are found.
- The third zone is the *contrasting zone*, containing word groups with low citation frequency but higher importance.
- The fourth zone is the *second periphery*, containing low frequency word groups that were considered less important.

Each word group was assigned to one of the four zones of the social representation map.

#### 2.3.2. Polarity Index 

To understand the implicit attitudes in this social representation, a polarity index (P) [21] was calculated for each word group.

(1) $$Polarity\;index\;\left(P\right)=\frac{\begin{array}{c} Number\;of\;positive\;words\;\\ in\;word\;group \end{array}-\begin{array}{c} Number\;of\;negative\;words\\ in\;word\;group \end{array}}{Total\;number\;of\;words\;in\;word\;group}$$

This index ranges from −1 to +1. A P value between +0.1 and +1 indicates that most of the words in that group are positive. If P is from −1 to −0.1, it indicates that most of the words in that group are considered negative. If P is from −0.04 to +0.04, positive and negative words are likely to be equal, indicating a neutral attitude. A polarity index was calculated for each word group, independently for (i) consumers and (ii) professionals.

## 3. Results

In total, 575 words were proposed either by consumers (*N* = 400, from which 7 words were then deleted as meaningless) or by professionals (*N* = 175). After the thematic analysis and the triangulation process, we obtained a total of 35 word groups, 24 of which were common to both sets of participants, while 6 groups were specific to consumers and 5 to professionals (Table 1). 

### 3.1. Structural Approach

#### 3.1.1. Social Representation Map for Consumers

**Figure 1:**
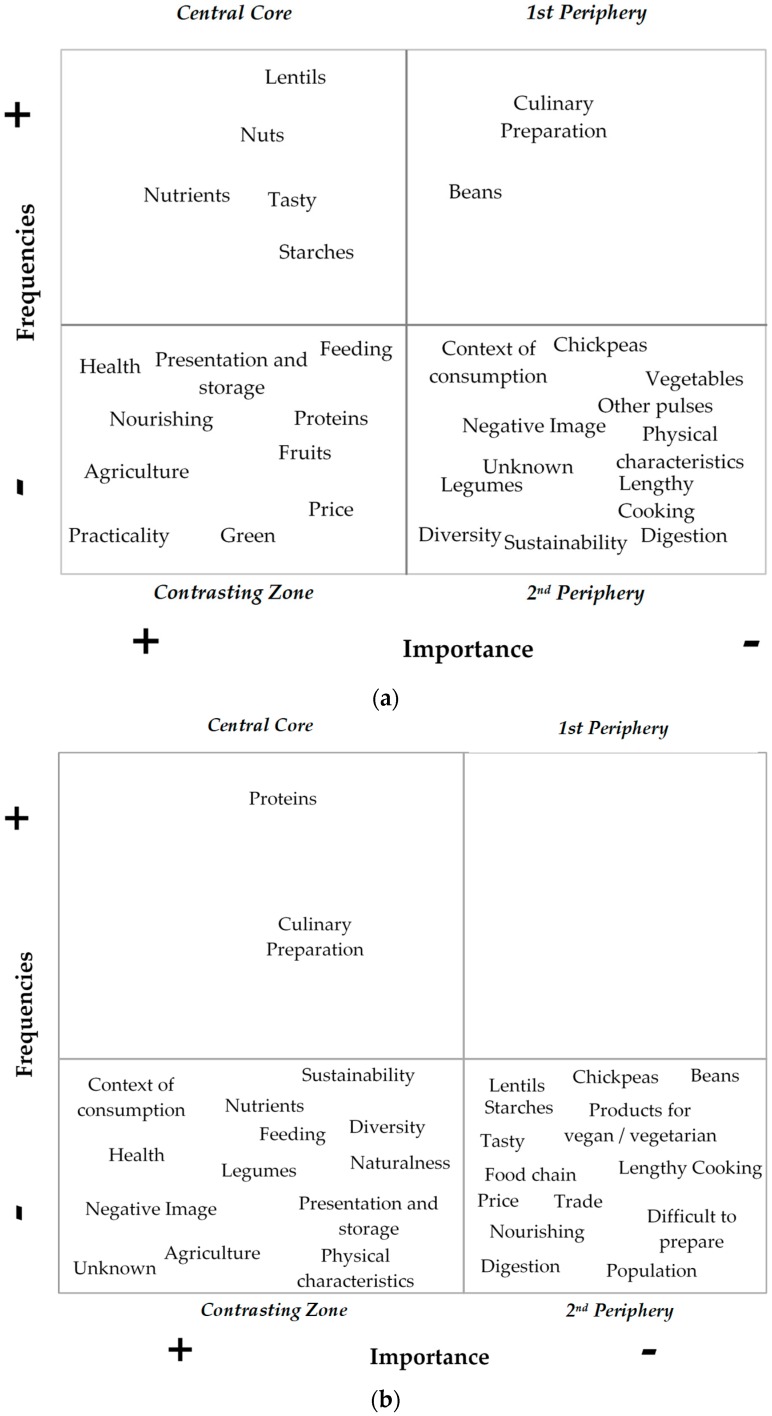
The social representation map for pulses among French nonvegetarian consumers (**a**), and for pulse food industry professionals (**b**).

Figure 1a shows the map for consumers. The cutoff point was 19 for frequency and 2.97 for importance. 

The central core contains the word groups *lentils, nutrients, tasty,* and *starches,* but, also, surprisingly, *nuts.* The first periphery contains only two word groups (*culinary preparation* and *beans)*. The contrasting zone and the second periphery are both richer and more varied. 

#### 3.1.2. Social Representation Map for Professionals

Figure 1b shows the map for pulse industry professionals. The cutoff point for frequency was 13, with 3.29 the cutoff point for importance. In the central core, there were only two word groups: *protein* and *culinary preparation.* Neither of these was common to the central core of the consumer map. Interestingly, the first periphery was empty. In the contrasting zone, we found word groups common to both consumers and professionals (*health, feeding*, *agriculture,* and *presentation and storage*), and others specific to professionals (*context of consumption*, *nutrients*, *sustainability*, *diversity*, *legumes*, and *naturalness*). Similarly, in the second periphery, some word groups were common to both consumers and professionals (*chickpeas*, *lengthy cooking*, and *digestion*). All the exemplars used by professionals are located in this zone (*lentils*, *chickpeas*, and *beans*).

### 3.2. Polarity Index 

**Figure 2:**
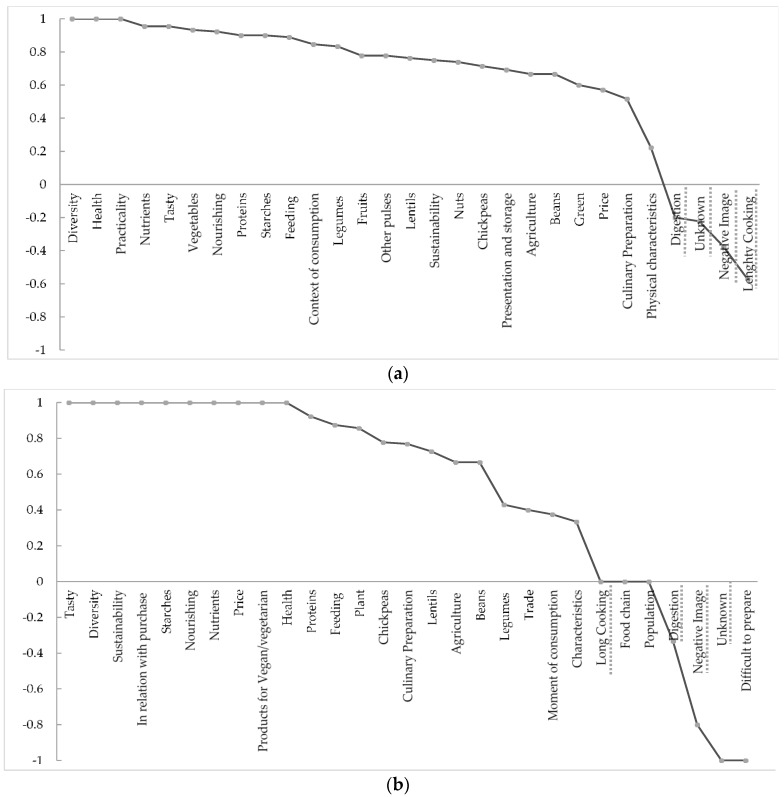
Polarity Index for French nonvegetarian consumers (**a**), and for professionals (**b**). The word groups underlined with a dotted line are common to both consumers and professionals, with either neutral or negative valence.

The polarity index was calculated for the word groups used by consumers (Figure 2a) and for those used by pulse industry professionals (Figure 2b). Most of the word groups have a positive valence for both sets of participants, reflecting a generally positive attitude towards pulses. Only a few word groups have negative valence; most of them (*digestion*, *unknown,* and *negative image*) are common to both consumers and professionals. Only one word group (*lengthy cooking)* is negative for consumers alone. The word group *difficult to cook* is negative for professionals, while *lengthy cooking, food chain,* and *population* are neutral for them. For consumers, none of the word groups was considered neutral. 

## 4. Discussion

The main goal of this study was to characterize the social representations of pulses for French nonvegetarian consumers and for professionals from the pulse industry in France. Results confirmed that although some word groups were shared, many were unique to one set of participants. Even when the representations for both sets of participants contained the same word groups, their positions on the maps were different. Social representations containing the same elements have been shown to be radically different when the relative positions of the elements are different [13]. Our study thus shows that the structure of social representations of pulses in France is different for consumers and for professionals: 

### 4.1. What is the social Representation of Pulses among French Nonvegetarian Consumers?

For the inductor “pulses”, the word group most frequently cited by consumers was “*lentils*”, while other pulses (e.g., *beans* or *chickpeas*) were either not frequently cited or not ranked highly. These spontaneous responses show that for French nonvegetarian consumers, lentils are the most prototypical pulses. This is in line with a previous study, in which French participants declared lentils to be the most liked and most frequently consumed pulse [9]. 

Items in the central core are known to resist change [29]. In this study, we observed that two other food groups (*nuts* and *starches*) were located in the central core, evidencing a strong association with pulses. In the French diet, pulses and starches are often considered to have the same function in a meal. As shown by Melendrez-Ruiz et al. (2019), French participants used pulses and starches as equivalent when composing a meal, often associating only one or the other with meat and vegetables [11]. Until 2016, French nutritional recommendations placed starches (i.e., bread, cereals, potatoes) and pulses in the same food category [30]. The strong association between starches and pulses in our study could be explained by consumers confusing the two food groups with regard to their properties and use.

The food group *nuts* was even more frequently cited and similarly ranked than *starches*, a surprising result potentially indicating that many participants in our study based their answers on semantic similarity. The inductor in French was “*légumes secs*”, the appropriate translation for “pulses”, but which translated literally would be “dry vegetables”. The word *nut* belongs to the category “*fruits secs*” in French, producing the literal translation “dry fruits”. Participants could therefore have focused on the adjective “*sec*” (“dry”), common to both expressions in French. This result reveals that strong semantic similarity between two food categories in a given language can induce overlapping representations of products belonging to different categories. 

Finally, the central core of the consumer map also contains two word groups related to pulse properties, *nutrients* and *tasty.* Pulses are known to contain many nutrients (e.g., iron, fiber, vitamins) that contribute to a healthy diet [31], and consumers in our study seemed to be aware of this fact. Interestingly, this intrinsic characteristic was mentioned alongside more individual, subjective perception (i.e., hedonicity).

The elements located in the first periphery are known to strengthen the meaning of the social representation [29]. In our study, this zone only contains two word groups: *beans* and *culinary preparation*. The frequent use of *beans*, in the same manner as *lentils* in the central core, indicates that consumers tend to use exemplars to confirm their understanding of the inductor. The use of *culinary preparation* is consistent with the results of our previous study, which showed that preparation is an important concern for consumers, which reduces their willingness to choose pulses [9]. The frequent citation of words related to culinary preparation in the present study reveals that this association between pulses and cooking is well anchored in consumers’ social representations.

The elements located in the contrasting zone are known to characterize consumer subgroups [29]. In our study, the great number and diversity of word groups in this zone reflect the broad range of unshared knowledge, beliefs, and attitudes regarding pulses among consumer subgroups. 

The second periphery contains more idiosyncratic elements, where individual differences can be observed [32]. All the word groups considered as negative by consumers (*negative image, unknown, lengthy cooking,* and *digestion*) were located in the second periphery. This is a key result, because the second periphery contains elements susceptible to change. Previous studies hypothesized that the low consumption of pulses in France could stem from negative attitudes toward pulses. It was reported, in particular, that pulses are considered as “the poor man’s meal” [2,33], not well known [3], require lengthy preparation, and may provoke unwanted digestive effects [2,3,6,33]. Our results do not confirm this hypothesis, since those attitudes are not present in the central core of social representations. This result means that negative attitudes toward pulses are not considered important and are not frequently observed among French nonvegetarian consumers but are linked only to a few individuals. As a consequence, they can be considered open to change.

### 4.2. What Is the Social Representation of Pulses among Pulse Industry Professionals in France?

With the inductor “pulses”, professionals mostly cited two word groups: *proteins* and *culinary preparation*. This reflects a more consensual point of view about pulses, but also a focus on the functional properties of pulses. The first periphery is surprisingly empty, confirming the strength of the representation for professionals.

The frequency of citation and high ranking of the word group *proteins* reflects the deeper understanding of the nutritional properties of pulses among professionals. Compared to consumers, they have more precise knowledge of pulses’ nutritional properties (*proteins* in the central core for professionals versus *nutrients* for consumers). 

Professionals did not cite any fruits, vegetables, or nuts, and only rarely starches. This result indicates that they share a clear definition of pulses and do not confuse them with other food groups that are semantically similar. For professionals, *lentils*, *beans*, and *chickpeas* are located in the second periphery, confirming that they did not need to cite exemplars to define what pulses are.

Surprisingly, a great diversity of word groups is observed in the second periphery, potentially reflecting the existence of subgroups with different representations [32], which could be explained by the profiles of the professionals who participated in this study. Different levels in the pulse supply chain are represented by specific terms. Some word groups are related to the agricultural sector (*naturalness*, *agriculture*, and *sustainability*), while others are related to the processing industry (*physical characteristics*) or the distribution sector (*context of consumption*, *nutrients*, *feeding*, and *presentation and storage*).

### 4.3. Discrepancies between the Representations of Consumers and Professionals

Several differences were observed between the social representations of consumers and of pulse industry professionals. One of the discrepancies evidenced by our study is that knowledge of the nutritional properties of pulses is more precise for professionals than for consumers, who need to cite exemplars to confirm the meaning of pulses. Most of the word groups in the central core of the consumer map are found in the second periphery of the professional map. For consumers, *tasty* is a stable fundamental component of their representation of pulses, whereas for professionals, this word group is idiosyncratic, revealing a meaningful difference between the two populations. The high number of citations and high ranking suggest that consumers appreciate the taste of pulses, associating specific organoleptic properties with them. Surprisingly, taste is less frequently mentioned by professionals, although it could be a potential lever to increase pulse consumption. The word group *negative image* is used differently by the two sets of participants. For consumers, it is located in the second periphery and has a polarity index of −0.4, whereas for professionals, it is located in the contrasting zone and has a polarity index of −0.8. This discrepancy indicates that professionals have slightly a more negative image of pulses than consumers have. 

An apparent paradox emerged from our results. Despite not knowing pulses very well, consumers do not have a negative image of them and even express a positive opinion about their taste. By contrast, professionals, who are supposed to know pulses well, do not mention their taste. This is unexpected, because professionals should consider taste as a functional property, like protein content, and use this characteristic to encourage consumption. Professionals even expressed a more negative opinion than consumers about pulses. Responses from professionals should be interpreted with caution: They may reflect both their personal attitudes and beliefs regarding pulses, and also their view of consumers’ beliefs and attitudes. In both cases, inaccurate communication about pulses centered on their protein content and preparation may be the result, although consumers would undoubtedly be more sensitive to arguments about the organoleptic properties of pulses. 

Finally, the word group *unknown* is found in the same zone (second periphery) for the two sets of participants, but with a polarity index of −0.2 for consumers and −1 for professionals. For professionals, lack of knowledge with regard to pulses represents a major disadvantage for their business.

### 4.4. What Could Be Done to Improve Communication about Pulses?

The differences between the social representations of consumers and professionals represent a barrier to pulse consumption, and communication could be a lever to overcome this. Both sets of participants need to be involved in communication strategies.

Professionals seem to place particular value on the high protein content of pulses and think this should be an important concern for consumers. Nonetheless, this opinion is not necessarily true: Consumers rarely cited the word group *proteins*, and even though they did often mention *nutrients*, the key element for them seems to be *tasty*. Consequently, due to this discrepancy, it will be difficult to produce consensual messages to communicate efficiently about the benefits of pulses. 

When questioned about pulses, consumers used some exemplars, indicating only vague and confused knowledge associated with this food group. It would be necessary to remove word groups such as *nuts* and *starches* from consumer representations to help with a clearer definition of pulses. Nevertheless, consumers cite positive qualities when they are questioned. Most of them show favorable beliefs and attitudes. Thus, there are no strong barriers to pulse consumption. A more efficient communication strategy would use more accessible words to communicate with consumers. However, communicating about “pulses” per se can be misunderstood by consumers. As long as the term “pulses” and pulse properties remain unclear, it will be difficult to communicate efficiently. There is a clear need to map out the terms and expressions used by consumers when referring to pulses. If public authorities and the pulse industry want to use the term “pulses”, educational programs and dietary recommendations should be adapted. Associating exemplars with the term “pulses” seems a necessary step, until a clearer social representation of pulses is reached. The problem of the indiscriminate use of the words “*légumes secs*” (pulses) and “*légumineuses*” (legumes) by French consumers must be addressed, because it may lead to confusion and misunderstanding. 

All the sectors involved in the pulse industry should work together to overcome this difficulty. Developing the relationship between stakeholders in the supply chain is essential for the meaningful diffusion of scientific knowledge in order to bring about changes in cultural practices [5].

### 4.5. Strengths and Limitations 

Applying the same methodology (the structural approach with polarity index calculation) to both sets of participants (consumers and professionals) provides a sound basis to assess the interplay between their social representations, attitudes, and beliefs with regard to pulses.

The small number of professionals from the pulse industry who participated in the study could be seen as a limiting factor, but it should be pointed out that this sector of activity employs relatively few people in France. With a larger number of professionals, it would be possible to form subgroups according to their main sector of activity (farmers/farming, transformation/processing, and distribution). Differences related to their main activity could thus be identified, leading to specific strategies for each sector in their communication with consumers. 

## 5. Conclusions

Our study highlighted new insights concerning social representations of pulses for French consumers and professionals from the French pulse industry. Differences were found in the social representations of these two groups, which were identified as a possible barrier to pulse consumption.

The main results revealed that consumers do not share a clear and stable social representation of pulses as a specific food group but tend to consider each type of pulse individually (e.g., beans, lentils, and chickpeas). The properties linked to pulses are mostly positive for consumers, and *tasty* was one of the major characteristics associated with pulses. By contrast, professionals’ answers were focused more on protein content and culinary preparation. This difference in focus has led to a communication breakdown between the two populations. 

In order to deliver a more efficient message to consumers about pulses, two steps are necessary. First, there is a need to map out the terms and expressions used by consumers when referring to pulses. These terms should be preferred, and any alternative should be clearly defined. Secondly, efforts to improve communication should involve all stakeholders in the pulse supply chain in France, as well as external bodies such as national authorities, schools, and associations. Their main focus should be on producing clear, precise, standardized messages about what pulses are. In addition, it is important to take into consideration the importance of taste, because taste is one of the main drivers for consumers. Overall, strengthening the relationship between consumers and professionals is essential in order to overcome the main barriers to pulse consumption while developing the best strategies to promote pulses. 

Efforts to achieve this goal would certainly be successful, because the few negative representations observed in our study were not stable and were shared by only a small number of people, indicating that such representations are ripe for change.

## Figures and Tables

**Table 1:** Frequency of use for each word group, for professionals and for consumers, with examples of words evoked for “pulses”. Italics for the word group name in French.

Word Group	Professionals (*n* = 35)	Consumers (*n* = 80)	Examples of Words Proposed by Participants	
Agriculture (*Agriculture*)	3	9	water, pesticide, planting, garden, seed	
Beans *(Haricots)*	4	27	beans, white beans, dried beans	
Chickpeas (*Pois Chiches*)	9	14	chickpeas	
Context of consumption (*Contexte de consommation*)	8	13	childhood, family dish, winter, sharing, meal, restaurant, sport	
Culinary Preparation (*Préparation culinaire*)	13	31	pressure-cooker, cooker, dish, dip, steam, recipe	
Difficult to prepare (*Préparation difficile*)	2	0	hard to cook	
Digestion (*Digestion*)	3	5	digestion, gas	
Diversity (*Diversité*)	10	4	diversity, variety	
Feeding (*Alimentation*)	8	18	food, eat, feeding	
Food chain (*Filière profesionnelle*)	4	0	profession, sector, competitively	
Fruits (*Fruits frais*)	0	9	apricots, bananas, grapes	
Green (*Vert*)	0	5	green	
Health (*Santé*)	8	17	beneficial, healthy	
Legumes (*Légumineuses*)	7	6	legumes, soya beans	
Lentils (*Lentilles*)	11	38	lentils, red lentils	
Lengthy Cooking (*Cuisson longue*)	1	7	lengthy preparation, lengthy cooking	
Naturalness (*Naturalité*)	7	0	plant-based, natural	
Negative Image (*Image négative*)	5	8	not good, insipid, not very popular, unattractive, not tasty	
Nourishing (*Nourrisant*)	1	13	appetite, energy, satiating, calorie	
Nutrients (*Nutriments*)	9	22	iron, nutritive, high in fiber, vitamins, balanced	
Nuts (*Fruits secs*)	0	23	almond, peanut, hazelnut, nut, pistachio	
Other pulses (*Autres légumes secs*)	0	9	split peas, flageolet beans	
Physical characteristics (*Caractéristiques physiques*)	3	9	brown, old, dehydrated, dry, solid	
Population (*Population*)	1	0	population	
Practicality (*Practicité*)	0	5	speed, flexibility, ease of preparation	
Presentation and storage (*Présentation et conservation*)	2	13	nonperishable, box, canned, convenient	
Price (*Prix*)	3	7	cost, economical, affordable	
Products for Vegans/Vegetarians (*Produits vegan/végétarien*)	3	0	vegan, vegetarian	
Proteins (*Protéines*)	26	10	proteins	
Starches (*Féculents*)	3	20	wheat, bulgur, potato, rice, quinoa	
Sustainability (*Durabilité*)	11	5	organic, environment, sustainable agriculture	
Tasty (*Bon*)	4	22	good, taste, flavor, delicious, fun	
Trade (*Commerce*)	5	0	import, worldwide, Italy, France	
Unknown (*Inconnu*)	1	9	little known, unknown	
Vegetables (*Légumes*)	0	15	carrots, spinach, green beans, salad	
Total	175	393 *		

* The 7 words deleted from the original total of 400 were: game, non-exclusive, exit, telephone, car, possibility, goldfish.
